# The Prognosis of Postural Albuminuria

**Published:** 1904-06-18

**Authors:** 


### THE PROGNOSIS OF POSTURAL ALBUMINURIA.

The discovery of albumen in the urine, even in the absence of all other evidences of disease, always gives rise to anxiety. Dr. G. A. Sutherland argues that there is one form of albuminuria, occurring during convalescence, induced solely by the adoption of the erect posture, and most probably traceable to an underlying condition of neurasthenia. He denies that this condition is associated either with a previous nephritis or with an active nephritis; also that postural albuminuria indicates any tendency to the development of kidney disease. In a series of 70 cases he has never seen the development of any evidence of organic renal disease, either in the examination of the urine or in the development of cardio-vascular or retinal changes. Postural albuminuria and organic renal disease he regards as entirely different affections. Hence he advises against the prescription for the former of the dietetic and other restrictions which are wisely adopted in cases of Bright's disease.

[1] Polyclinic, June 1904.

